# (*E*)-2-(4-Nitro­benzyl­ideneamino)benzamide

**DOI:** 10.1107/S160053680902203X

**Published:** 2009-06-13

**Authors:** Shu-Liang Wang, Ke Yang, Xiang-Shan Wang, Shu-Jiang Tu

**Affiliations:** a School of Chemistry and Chemical Engineering, Xuzhou Normal University, Xuzhou Jiangsu 221116, People’s Republic of China

## Abstract

The title compound, C_14_H_11_N_3_O_3_, adopts an *E* conformation, with a dihedral angle of 41.8 (1) ° between the mean planes of the two benzene rings. One of the amino H atoms forms an intra­molecular hydrogen bond with the amide N atom, while the other H atom forms an inter­molecular hydrogen bond with the carbonyl O atom of an adjacent mol­ecule, forming dimers about inversion centers. A non-classical inter­molecular C—H⋯O hydrogen bond also links adjacent mol­ecules into dimers.

## Related literature

For Schiff bases complexes with metal ions, see: Kannan & Ramesh (2006 ▶); Lv *et al.* (2006 ▶); Maurya *et al.* (2006 ▶); Parekh *et al.* (2006 ▶); Vanco *et al.* (2004 ▶).
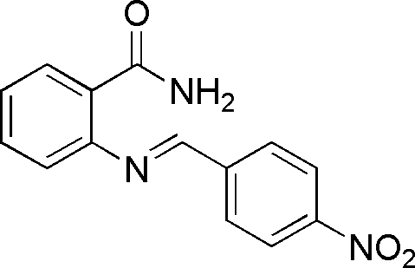


## Experimental

### 

#### Crystal data

C_14_H_11_N_3_O_3_
*M* *_r_* = 269.26
Monoclinic, 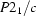

*a* = 7.3863 (2) Å
*b* = 12.2657 (3) Å
*c* = 14.1414 (4) Å
β = 97.248 (1)°
*V* = 1270.95 (6) Å^3^
*Z* = 4
Mo *K*α radiation
μ = 0.10 mm^−1^
*T* = 296 K
0.45 × 0.29 × 0.16 mm

#### Data collection

Bruker SMART CCD area-detector diffractometer
Absorption correction: none
9505 measured reflections
2278 independent reflections
1809 reflections with *I* > 2σ(*I*)
*R*_int_ = 0.026

#### Refinement

*R*[*F* ^2^ > 2σ(*F* ^2^)] = 0.034
*wR*(*F* ^2^) = 0.093
*S* = 1.04
2278 reflections
190 parameters
H atoms treated by a mixture of independent and constrained refinement
Δρ_max_ = 0.14 e Å^−3^
Δρ_min_ = −0.14 e Å^−3^

### 

Data collection: *SMART* (Bruker, 2001 ▶); cell refinement: *SAINT* (Bruker, 2001 ▶); data reduction: *SAINT*; program(s) used to solve structure: *SHELXS97* (Sheldrick, 2008 ▶); program(s) used to refine structure: *SHELXL97* (Sheldrick, 2008 ▶); molecular graphics: *SHELXTL* (Sheldrick, 2008 ▶); software used to prepare material for publication: *SHELXTL*.

## Supplementary Material

Crystal structure: contains datablocks global, I. DOI: 10.1107/S160053680902203X/pv2166sup1.cif

Structure factors: contains datablocks I. DOI: 10.1107/S160053680902203X/pv2166Isup2.hkl

Additional supplementary materials:  crystallographic information; 3D view; checkCIF report

## Figures and Tables

**Table 1:** Hydrogen-bond geometry (Å, °)

*D*—H⋯*A*	*D*—H	H⋯*A*	*D*⋯*A*	*D*—H⋯*A*
C13—H13*A*⋯O2^i^	0.93	2.44	3.1903 (19)	138
N1—H1*B*⋯O3^ii^	0.904 (18)	2.059 (19)	2.9581 (17)	173.3 (15)
N1—H1*A*⋯N2	0.877 (18)	1.999 (18)	2.7027 (18)	136.4 (15)

Symmetry codes: (i) 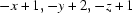; (ii) 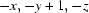.

**Fig. 1.**
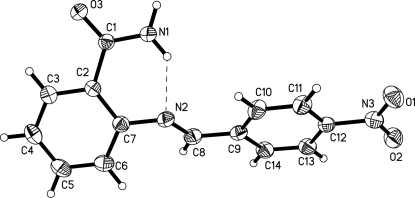
The molecular structure of (I) showing 50% probability of displacement ellipsoids and the atom-numbering scheme.

**Fig. 2.**
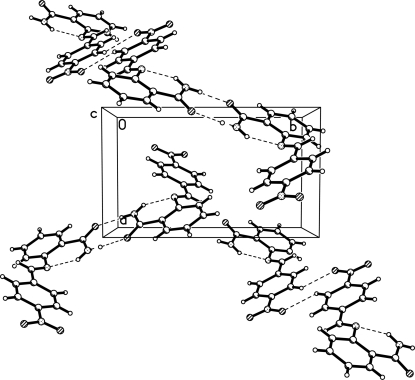
The molecular packing diagram showing the hydrogen-bonding network in the crystal for (I).

## supplementary crystallographic information

### Comment

Schiff bases are well known as ligands for many metal ions, such as copper(II) (Vanco *et al.*, 2005), Vanadium(IV, V) (Maurya *et al.*, 2006) and ruthenium(III) (Kannan & Ramesh, 2006). Some of metal Schiff base complexes possess biological activities. For example, it was reported that oxovanadium(IV) complexes with Schiff bases had antifungal activity (Parekh *et al.*, 2006), cobalt(II) and copper(II) complexes of valine-derived Schiff bases possessed antimicrobial activity (Lv *et al.*, 2006). We have synthesized the title compound, (I), by the reaction of 4-nitrobenzaldehyde, 2-aminobenzamide in an ionic liquid at room temperature which is reported in this article.

The X-ray crystal structure determination indicates that the title compound adopts an E-configuration (Fig. 1). The plane defined as the atoms of C7, C8, C9, N2 and H8A is nearly parallel to the benzene ring (C9—C14), forming a dihedral angle of 0.8 (1)°. The dihedral angle between the basal plane (atoms C7, C8, C9, N2 and H8A) and  the other benzene ring (C2—C7) is 41.1 (1)°. the  benzene rings make a dihedral angle of 41.8 (1)°.

The classical (N—H···O) and unclassical (C—H···O) hydrogen bonds are present in the crystal structure of (I) (Table 1). One of the hydrogen atoms (H1A) on the amino group forms an intra-molecular hydrogen bond (N1—H1A···N2) with the atom N2, while the other hydrogen atom (H1B) forms an inter-molecular hydrogen bond of the type N—H···O with the atom O3 in the adjacent molecule, forming dimmers. An unclassical intermolecular hydrogen bond (C13—H13A···O2) also links the adjacent molecules into dimmers (Fig. 2). The above inter-molecular hydrogen bonds link the molecules into polymers.

### Experimental

The title compound, (I), was prepared by the reaction of 4-nitrobenzaldehyde (2 mmol, 0.302 g) and 2-aminobenzamide (2 mmol, 0.272 g) in an ionic liquid of [Bmim]Br (Bmim = 1-butyl-3-methylimidazolium) (2 ml) at 353 K; m.p. 457–458 K. The single crystals of (I) suitable for X-ray diffraction were obtained by slow evaporation of an ethanol solution.

### Refinement

The H atoms bonded to C atoms were calculated geometrically and refined as riding, with C—H = 0.93 Å while the amino H-atoms were allowed to refine; *U*_iso_(H) = 1.2*U*_eq_(parent atom).

### Crystal data

**Table:**

C_14_H_11_N_3_O_3_	*F*(000) = 560
*M**_r_* = 269.26	*D*_x_ = 1.407 Mg m^−^^3^
Monoclinic, *P*2_1_/*c*	Melting point = 457–458 K
Hall symbol: -P 2ybc	Mo *K*α radiation, λ = 0.71073 Å
*a* = 7.3863 (2) Å	Cell parameters from 3148 reflections
*b* = 12.2657 (3) Å	θ = 2.2–25.8°
*c* = 14.1414 (4) Å	µ = 0.10 mm^−^^1^
β = 97.248 (1)°	*T* = 296 K
*V* = 1270.95 (6) Å^3^	Block, yellow
*Z* = 4	0.45 × 0.29 × 0.16 mm

### Data collection

**Table:**

Bruker SMART CCD area-detector diffractometer	1809 reflections with *I* > 2σ(*I*)
Radiation source: fine-focus sealed tube	*R*_int_ = 0.026
graphite	θ_max_ = 25.2°, θ_min_ = 2.2°
φ and ω scans	*h* = −8→8
9505 measured reflections	*k* = −14→14
2278 independent reflections	*l* = −16→16

### Refinement

**Table:**

Refinement on *F*^2^	Secondary atom site location: difference Fourier map
Least-squares matrix: full	Hydrogen site location: inferred from neighbouring sites
*R*[*F*^2^ > 2σ(*F*^2^)] = 0.034	H atoms treated by a mixture of independent and constrained refinement
*wR*(*F*^2^) = 0.093	*w* = 1/[σ^2^(*F*_o_^2^) + (0.0436*P*)^2^ + 0.1992*P*] where *P* = (*F*_o_^2^ + 2*F*_c_^2^)/3
*S* = 1.04	(Δ/σ)_max_ < 0.001
2278 reflections	Δρ_max_ = 0.14 e Å^−^^3^
190 parameters	Δρ_min_ = −0.14 e Å^−^^3^
0 restraints	Extinction correction: *SHELXL97* (Sheldrick, 2008), Fc^*^=kFc[1+0.001xFc^2^λ^3^/sin(2θ)]^-1/4^
Primary atom site location: structure-invariant direct methods	Extinction coefficient: 0.046 (3)

### Special details

**Table:**

Geometry. All e.s.d.'s (except the e.s.d. in the dihedral angle between two l.s. planes) are estimated using the full covariance matrix. The cell e.s.d.'s are taken into account individually in the estimation of e.s.d.'s in distances, angles and torsion angles; correlations between e.s.d.'s in cell parameters are only used when they are defined by crystal symmetry. An approximate (isotropic) treatment of cell e.s.d.'s is used for estimating e.s.d.'s involving l.s. planes.
Refinement. Refinement of *F*^2^ against ALL reflections. The weighted *R*-factor *wR* and goodness of fit *S* are based on *F*^2^, conventional *R*-factors *R* are based on *F*, with *F* set to zero for negative *F*^2^. The threshold expression of *F*^2^ > σ(*F*^2^) is used only for calculating *R*-factors(gt) *etc*. and is not relevant to the choice of reflections for refinement. *R*-factors based on *F*^2^ are statistically about twice as large as those based on *F*, and *R*- factors based on ALL data will be even larger.

### Fractional atomic coordinates and isotropic or equivalent isotropic displacement parameters (Å^2^)

**Table:**

	*x*	*y*	*z*	*U*_iso_*/*U*_eq_	
N2	0.29424 (15)	0.81888 (9)	0.04882 (9)	0.0480 (3)	
O2	0.66701 (16)	0.86265 (9)	0.54680 (8)	0.0669 (3)	
O3	−0.03944 (14)	0.58880 (8)	−0.10923 (7)	0.0556 (3)	
C9	0.39628 (18)	0.86357 (11)	0.21085 (11)	0.0459 (4)	
C2	0.10787 (17)	0.76078 (11)	−0.09651 (10)	0.0413 (3)	
C1	0.06905 (17)	0.64827 (11)	−0.06033 (10)	0.0412 (3)	
N3	0.66097 (17)	0.79210 (10)	0.48528 (10)	0.0525 (3)	
C12	0.56915 (18)	0.81749 (11)	0.38992 (10)	0.0445 (4)	
C7	0.20752 (18)	0.84286 (11)	−0.04369 (11)	0.0445 (3)	
N1	0.15232 (19)	0.61634 (11)	0.02351 (10)	0.0536 (4)	
C8	0.30111 (19)	0.88799 (12)	0.11590 (11)	0.0503 (4)	
H8A	0.2447	0.9554	0.1046	0.060*	
C14	0.3976 (2)	0.93889 (11)	0.28377 (11)	0.0521 (4)	
H14A	0.3381	1.0053	0.2721	0.063*	
C13	0.4860 (2)	0.91691 (11)	0.37357 (11)	0.0518 (4)	
H13A	0.4892	0.9684	0.4220	0.062*	
C3	0.0323 (2)	0.78476 (12)	−0.18910 (11)	0.0497 (4)	
H3A	−0.0348	0.7314	−0.2248	0.060*	
C11	0.5690 (2)	0.74035 (12)	0.31915 (13)	0.0540 (4)	
H11A	0.6261	0.6734	0.3318	0.065*	
C10	0.4840 (2)	0.76367 (12)	0.23011 (12)	0.0552 (4)	
H10A	0.4844	0.7124	0.1817	0.066*	
O1	0.72849 (17)	0.70200 (9)	0.49945 (9)	0.0717 (4)	
C4	0.0538 (2)	0.88540 (14)	−0.22967 (12)	0.0592 (4)	
H4A	0.0017	0.8994	−0.2918	0.071*	
C5	0.1527 (2)	0.96491 (14)	−0.17769 (13)	0.0645 (5)	
H5A	0.1680	1.0329	−0.2047	0.077*	
C6	0.2289 (2)	0.94391 (12)	−0.08568 (13)	0.0590 (4)	
H6A	0.2957	0.9981	−0.0510	0.071*	
H1A	0.226 (2)	0.6618 (14)	0.0573 (13)	0.067 (5)*	
H1B	0.121 (2)	0.5508 (15)	0.0458 (11)	0.060 (5)*	

### Atomic displacement parameters (Å^2^)

**Table:**

	*U*^11^	*U*^22^	*U*^33^	*U*^12^	*U*^13^	*U*^23^
N2	0.0529 (7)	0.0391 (6)	0.0509 (8)	−0.0056 (5)	0.0025 (6)	−0.0035 (6)
O2	0.0898 (8)	0.0617 (7)	0.0489 (7)	−0.0009 (6)	0.0074 (6)	−0.0088 (6)
O3	0.0673 (6)	0.0468 (6)	0.0495 (7)	−0.0128 (5)	−0.0050 (5)	−0.0018 (5)
C9	0.0473 (7)	0.0400 (7)	0.0507 (9)	−0.0063 (6)	0.0079 (6)	−0.0063 (7)
C2	0.0418 (7)	0.0405 (7)	0.0421 (8)	0.0023 (5)	0.0079 (6)	0.0007 (6)
C1	0.0440 (7)	0.0399 (7)	0.0404 (8)	−0.0003 (6)	0.0076 (6)	−0.0039 (6)
N3	0.0589 (7)	0.0471 (7)	0.0530 (9)	−0.0034 (6)	0.0135 (6)	−0.0007 (7)
C12	0.0464 (7)	0.0428 (8)	0.0452 (9)	−0.0052 (6)	0.0091 (6)	−0.0023 (7)
C7	0.0477 (7)	0.0388 (7)	0.0472 (9)	0.0022 (6)	0.0066 (6)	0.0012 (6)
N1	0.0678 (8)	0.0412 (7)	0.0484 (8)	−0.0137 (6)	−0.0058 (7)	0.0056 (6)
C8	0.0544 (8)	0.0396 (7)	0.0568 (10)	−0.0013 (6)	0.0067 (7)	−0.0037 (7)
C14	0.0666 (9)	0.0358 (7)	0.0544 (10)	0.0019 (6)	0.0098 (8)	−0.0051 (7)
C13	0.0696 (9)	0.0390 (8)	0.0482 (10)	−0.0030 (7)	0.0130 (8)	−0.0091 (7)
C3	0.0507 (8)	0.0529 (8)	0.0456 (9)	0.0022 (6)	0.0066 (7)	0.0024 (7)
C11	0.0569 (8)	0.0431 (8)	0.0610 (10)	0.0080 (6)	0.0041 (7)	−0.0095 (7)
C10	0.0620 (9)	0.0466 (8)	0.0553 (11)	0.0055 (7)	0.0014 (8)	−0.0167 (7)
O1	0.0907 (8)	0.0550 (7)	0.0685 (8)	0.0134 (6)	0.0062 (7)	0.0070 (6)
C4	0.0638 (9)	0.0637 (10)	0.0506 (10)	0.0091 (8)	0.0089 (8)	0.0151 (8)
C5	0.0774 (11)	0.0480 (9)	0.0693 (12)	0.0043 (8)	0.0140 (9)	0.0188 (9)
C6	0.0702 (10)	0.0384 (8)	0.0683 (12)	−0.0041 (7)	0.0080 (8)	0.0019 (8)

### Geometric parameters (Å, °)

**Table:**

N2—C8	1.2684 (19)	N1—H1A	0.877 (18)
N2—C7	1.4131 (19)	N1—H1B	0.904 (18)
O2—N3	1.2238 (16)	C8—H8A	0.9300
O3—C1	1.2299 (15)	C14—C13	1.379 (2)
C9—C14	1.384 (2)	C14—H14A	0.9300
C9—C10	1.397 (2)	C13—H13A	0.9300
C9—C8	1.466 (2)	C3—C4	1.379 (2)
C2—C3	1.389 (2)	C3—H3A	0.9300
C2—C7	1.4053 (19)	C11—C10	1.365 (2)
C2—C1	1.5118 (19)	C11—H11A	0.9300
C1—N1	1.3241 (19)	C10—H10A	0.9300
N3—O1	1.2186 (16)	C4—C5	1.375 (2)
N3—C12	1.4645 (19)	C4—H4A	0.9300
C12—C13	1.372 (2)	C5—C6	1.375 (2)
C12—C11	1.377 (2)	C5—H5A	0.9300
C7—C6	1.392 (2)	C6—H6A	0.9300
			
C8—N2—C7	121.60 (12)	C13—C14—C9	120.91 (14)
C14—C9—C10	118.75 (14)	C13—C14—H14A	119.5
C14—C9—C8	120.25 (13)	C9—C14—H14A	119.5
C10—C9—C8	120.99 (13)	C12—C13—C14	118.60 (13)
C3—C2—C7	118.09 (13)	C12—C13—H13A	120.7
C3—C2—C1	116.21 (12)	C14—C13—H13A	120.7
C7—C2—C1	125.67 (13)	C4—C3—C2	121.88 (14)
O3—C1—N1	121.61 (13)	C4—C3—H3A	119.1
O3—C1—C2	119.27 (12)	C2—C3—H3A	119.1
N1—C1—C2	119.12 (12)	C10—C11—C12	118.97 (14)
O1—N3—O2	123.15 (14)	C10—C11—H11A	120.5
O1—N3—C12	118.47 (13)	C12—C11—H11A	120.5
O2—N3—C12	118.38 (12)	C11—C10—C9	120.79 (14)
C13—C12—C11	121.95 (14)	C11—C10—H10A	119.6
C13—C12—N3	119.32 (13)	C9—C10—H10A	119.6
C11—C12—N3	118.73 (13)	C5—C4—C3	119.60 (15)
C6—C7—C2	119.41 (14)	C5—C4—H4A	120.2
C6—C7—N2	121.19 (13)	C3—C4—H4A	120.2
C2—C7—N2	119.27 (12)	C4—C5—C6	119.97 (15)
C1—N1—H1A	119.1 (12)	C4—C5—H5A	120.0
C1—N1—H1B	117.8 (10)	C6—C5—H5A	120.0
H1A—N1—H1B	122.9 (15)	C5—C6—C7	121.04 (15)
N2—C8—C9	121.19 (13)	C5—C6—H6A	119.5
N2—C8—H8A	119.4	C7—C6—H6A	119.5
C9—C8—H8A	119.4		
			
C3—C2—C1—O3	−7.15 (18)	C10—C9—C14—C13	1.0 (2)
C7—C2—C1—O3	170.60 (13)	C8—C9—C14—C13	179.76 (13)
C3—C2—C1—N1	173.09 (13)	C11—C12—C13—C14	1.0 (2)
C7—C2—C1—N1	−9.2 (2)	N3—C12—C13—C14	−178.97 (12)
O1—N3—C12—C13	177.42 (13)	C9—C14—C13—C12	−1.6 (2)
O2—N3—C12—C13	−2.95 (19)	C7—C2—C3—C4	0.5 (2)
O1—N3—C12—C11	−2.57 (19)	C1—C2—C3—C4	178.39 (13)
O2—N3—C12—C11	177.05 (13)	C13—C12—C11—C10	0.1 (2)
C3—C2—C7—C6	−0.8 (2)	N3—C12—C11—C10	−179.88 (13)
C1—C2—C7—C6	−178.46 (13)	C12—C11—C10—C9	−0.7 (2)
C3—C2—C7—N2	−176.60 (12)	C14—C9—C10—C11	0.2 (2)
C1—C2—C7—N2	5.7 (2)	C8—C9—C10—C11	−178.58 (14)
C8—N2—C7—C6	41.8 (2)	C2—C3—C4—C5	0.0 (2)
C8—N2—C7—C2	−142.45 (14)	C3—C4—C5—C6	−0.2 (2)
C7—N2—C8—C9	−178.08 (12)	C4—C5—C6—C7	−0.1 (3)
C14—C9—C8—N2	−178.12 (14)	C2—C7—C6—C5	0.6 (2)
C10—C9—C8—N2	0.6 (2)	N2—C7—C6—C5	176.34 (14)

### Hydrogen-bond geometry (Å, °)

**Table:**

*D*—H···*A*	*D*—H	H···*A*	*D*···*A*	*D*—H···*A*
C13—H13A···O2^i^	0.93	2.44	3.1903 (19)	138
N1—H1B···O3^ii^	0.904 (18)	2.059 (19)	2.9581 (17)	173.3 (15)
N1—H1A···N2	0.877 (18)	1.999 (18)	2.7027 (18)	136.4 (15)

Symmetry codes: (i) −*x*+1, −*y*+2, −*z*+1; (ii) −*x*, −*y*+1, −*z*.
